# Changes in adolescents’ sleep during COVID-19 outbreak reveal the inadequacy of early morning school schedules

**DOI:** 10.5935/1984-0063.20200127

**Published:** 2022

**Authors:** Jefferson Souza Santos, Fernando Mazzilli Louzada

**Affiliations:** Federal University of Paraná, Department of Physiology - Curitiba - Paraná - Brazil.

**Keywords:** Adolescent, Sleep, Coronavirus Infections

## Abstract

**Objectives:**

The forced closure of schools during the COVID-19 outbreak imposed on adolescents a new reality of home-schooling. This new situation has affected adolescent sleep patterns due to the absence of the pressure to wake up earlier induced by school times during pandemic. This study aimed to investigate the changes in sleep and napping habits in Brazilian adolescents during the COVID-19 outbreak.

**Methods:**

A sample of 259 high school adolescents (mean age = 15.5 years) reported sleep and napping habits by means validated questionnaires in both baseline year (March-June 2019) and during COVID-19 lockdown (July 2020).

**Results:**

The tendency to eveningness was higher and daytime sleepiness was reduced during the social isolation. Time in bed (TIB) increased by more than 2 hours and sleep onset time was delayed during the pandemic. More adolescents reported getting enough TIB during the pandemic. Moreover, sleepiness during remote classes was reduced compared to that reported during traditional classes one year before. The nap habit decreased during the pandemic compared to the baseline year.

**Discussion:**

The lack of early wake-up pressure to attend school in the morning could explain the sleep improvements perceived during the COVID-19 outbreak. Therefore, parents, educators, and policy makers need to discuss more feasible school times for adolescents in order to implement these changes as soon as returning to presential/hybrid learning.

## INTRODUCTION

Since the World Health Organization (WHO) declared COVID-19 as a public health emergency of international concern, countries around the world have adopted interventions to control the epidemic^1^. Non-pharmaceutical interventions oriented by WHO have been implemented, such as isolation, quarantining, social distancing, and community containment^1^. People worldwide needed to adapt their lives in a new stay- at-home situation what has impacted sleep and mental health^2,3,4^. Many countries were forced to close schools and adopt home-schooling, which have generated negative consequences to mental health^5,6^ and changes in adolescents’ sleep patterns^7,8,9^ - for example, a delay in sleep timing^8^.

Adolescence is considered a striking age range of physical and mental changes, which reinforces the essential role of adequate sleep in maintaining health and well-being^10^. Nevertheless, adolescents face chronic sleep restriction and some factors contribute to this insufficient sleep: social, caused by early school starting times; bioregulatory, responsible for delayed circadian rhythms and slowed sleep pressure; and psychosocial, since they experiment later nighttime screen use and more bedtime autonomy^11,12^. However, a special concern has been raised about the detrimental effects of the early school starting times in adolescent sleep.

The interaction between circadian and homeostatic changes (inducing delayed bedtimes) and early school starting times (inducing earlier wake times) is one of the main factors affecting adolescents’ sleep^12^. The need to wake up early for the morning shift students triggers social jet lag^13^, poor academic performance^14^, emotional impairments, and risk-taking behaviors^15^. These consequences are usually seen in countries where most of public high schools adopt an early morning shift. Therefore, students need to face this chronic sleep restriction, which results in the adoption of different strategies such as the consumption of caffeine, energy drink intake, and napping behavior to alleviate the detrimental effects of poor sleep time^16,17,18^.

Napping is a frequent practice among adolescents to reduce daytime sleepiness and cognitive deficits experienced after sleep restriction^19^. In some countries, the nap habit is defined as a healthy lifestyle with schools providing specific times of the day to nap opportunity from primary to high school grades^20^. Several studies have pointed out that napping provides benefts to sustained attention^21^, concentration^22^, to consolidate declarative and procedural memory^23,24,25^, and to executive function improvements^26,27,28^. On the other hand, prolonged naps have the potential to generate sleep inertia^29^, impairing physical^30^ and cognitive performances^29^, affecting subsequent nocturnal sleep^17,31^.

Therefore, the aim of this study was to investigate the possible changes in sleep and napping habits in Brazilian adolescents during the COVID-19 outbreak. The authors hypothesized that the social isolation would decrease daytime sleepiness due to the nocturnal sleep improvements. In addition, changes in napping behavior would be expected during the COVID-19 lockdown.

## MATERIAL AND METHODS

### Participants

The sample was composed of 259 public high school adolescents (mean age = 15.5 years; 76.1% girls) studying in the morning shift (starting time: 7:30 a.m.). The selection of the schools was based on randomly choice among the whole of nine sectors divided by the State Department of Education (SEED-PR) located in the city of Curitiba, Brazil (25°25′S, 49°16′W). At least one school per sector was chosen, encompassing only those located in the urban area. The legal permission to execute this study in the scholar field was approved by the SEED-PR.

### Procedures

The first data collection (baseline year) occurred in March-June 2019 followed by a recollection in the subsequent year (pandemic year) in July 2020. The survey was applied in conventional morning school activity-time inside classrooms in the baseline year. Firstly, the study aims and data collection processes were described to students. Secondly, the non-obligation to participate in the survey was clarifed. After the student informed assenting, the consent provided by parents or legally responsible was required. On the following day, to ensure better comprehension, a brief explanation of the survey was presented, as well as instructions on how to successfully fulfll the questionnaires that were available on tablet devices. One year after the first participation, the same students were contacted via *WhatsApp* and asked to answer the same questionnaires. The data collected during the pandemic year occurred after school closure by means of a *Google Forms* survey.

The students provided demographic information and fulfilled the pediatric daytime sleepiness scale (PDSS), the morningness-eveningness scale (M/E), and the Pittsburgh sleep quality index (PSQI). The napping habits were asked by a single question (“*Do you usually take naps during the day?*”). The exclusion criteria covered self-reports about sleep disturbances, health problems, and medication use with the potential to infuence the sleep-wake cycle.

### Measures

#### Pediatric Daytime Sleepiness Scale (PDSS)

The translated and validated version to Brazilian adolescents of the PDSS has been showed a reliable instrument to evaluate excessive daytime sleepiness in children and adolescents^32^. This scale evaluates sleepiness through 8 questions with 5-point Likert-type options from never (0) to always (5). Through the sum of points, scores were ranged from 0 (low sleepiness) to 32 (high sleepiness) with higher scores indicating greater daytime sleepiness. Internal consistency of PDSS was acceptable to this sample (Cronbach’s alpha = 0.64).

### Morningness/eveningness scale (M/E)

Initially proposed by Carskadon et al. (1993)^33^, the M/E scale measures the tendency to morningness or eveningness in adolescents. This version was also translated and validated to Brazilian adolescents^34^. Questions about time-of-day to perform activities (e.g., sleep/wake times, physical exercise, leisure, and scholar activities) compose the whole of 10 multiple-choice questions providing a final score by means of the sum of answers. The maximum score is 43 - higher values indicate a morningness tendency whereas lower values indicate eveningness tendency. The M/E showed adequate internal consistency to this sample (Cronbach’s alpha = 0.76).

### Pittsburgh Sleep Quality Index (PSQI)

The measurement of sleep quality was based on the Brazilian version validated for adolescents by Passos et al. (2017)^35^. PSQI is composed of 19 questions grouped into seven components: (C1) subjective sleep quality, (C2) sleep latency, (C3) sleep duration, (C4) habitual sleep efficiency, (C5) sleep disturbances, (C6) use of sleep medication, and (C7) daytime dysfunction. The factor analysis performed better excluding the component 6 (use of sleep medication) of PSQI to adolescents’ samples^35^. In addition, PSQI provides self-report bedtime and wakeup time behaviors. A global score is generated by the sum of these components indicating worse sleep quality in higher scores. Poor sleepers are classifed with scores greater than 5 points in PSQI^36^. The internal consistency analysis indicated adequate reliability of PSQI to this sample (Cronbach’s alpha = 0.68).

### Statistical analysis

The continuous variables were expressed as mean and standard deviation (SD) whereas the categorical variable was described as frequency distribution. To investigate the normality of the data a Kolmogorov-Smirnov test was performed. The differences between baseline (2019) and pandemic (2020) years for each variable were analyzed through linear mixed models (LMM) to control for potential confounders. The time of the data collection (baseline or pandemic year) and biological sex were used as factors (fixed effects) and age as covariate (random effect). The McNemar’s test was used to compare categorical variables among the years. An effect size test (Cohen’s *d*) was performed for all the comparisons of means. The analysis was executed using both SPSS, version 25, (IBM Corp., Armonk, N Y, U.S.A.) and Python version 3.7 (Spyder IDE). The significance level was defined by *p*<0.05.

### Ethical aspects

This study was approved by the Local Research Ethics Committee (CEP – Universidade Federal do Paraná, Brazil), number of process 72937617.1.0000.0102, authorized in October 2017, in compliance with the declaration of Helsinki.

## RESULTS

The demographic characterization of the sample is showed in Table 1. The mean age in the baseline year was 15.5±1.11 while during lockdown (pandemic year) the mean age was 16.8±1.15. The adolescents during COVID-19 pandemic reported enough time in bed (TIB) more frequently than one year before (*p*<0.001, d=0.50). Sleepiness showed a decrease during remote classes at home compared to sleepiness in the classroom one year before (*p*<0.001, d=0.68). Daytime sleepiness scored by PDSS decreased during pandemics (*p*<0.001, d=0.40), despite the fact that sleep quality did not differ (*p*=0.92, d=0.01). The students showed a higher tendency to eveningness during the pandemic year compared to baseline (*p*<0.001, d=0.60).

**Table 1.** Demographic characterization from years in baseline and pandemic groups.

	Baseline year	Pandemic year	Estimates	SD		LMM Fixed effects		95% CI
	Mean ± SD	Mean ± SD			t	p	d	
Enough TIB*	1.29 ± 1.14	1.75 ± 1.19	-0.46	0.08	-5.72	<0.001	0.50	-0.63 to -0.31
SC/SRC**	2.31 ± 1.06	1.56 ± 1.43	0.75	0.10	7.68	<0.001	0.68	0.55 to 0.93
PDSS	17.99 ± 4.87	16.58 ± 5.05	1.39	0.31	4.53	<0.001	0.40	0.79 to 2.01
M/E	25.14 ± 5.43	22.94 ± 6.07	2.20	0.32	6.82	<0.001	0.60	1.56 to 2.83
PSQI (score)***	6.56 ± 2.83	6.58 ± 3.00	-0.02	0.19	-0.10	0.92	0.01	-0.39 to 0.36
(C1)	1.33 ± 0.72	1.36 ± 0.83	-0.03	0.06	-0.47	0.64	0.04	-0.14 to 0.08
(C2)	1.41 ± 0.95	1.54 ± 0.98	-0.13	0.06	-2.13	0.03	0.19	-0.27 to -0.01
(C3)	0.90 ± 0.84	0.37 ± 0.70	0.53	0.06	9.10	<0.001	0.80	0.42 to 0.65
(C4)	0.25 ± 0.60	0.79 ± 1.04	-0.54	0.07	-7.98	<0.001	0.70	-0.67 to -0.40
(C5)	1.19 ± 0.61	1.16 ± 0.59	0.03	0.04	0.91	0.36	0.08	-0.04 to 0.11
(C7)	1.48 ± 0.80	1.36 ± 0.75	0.12	0.06	2.00	0.046	0.18	0.01 to 0.23

**Notes:** *Both score in enough TIB and sleepiness in the classroom/remote classes is from 0 (never) to 4 (always); ^**^Sleepiness in the classroom/sleepiness in remote classes (SC/SRC). This variable was extracted by the first question of pediatric daytime sleepiness scale (PDSS): “*How often do you fall asleep or get drowsy during class periods?*”. Sleepiness in the classroom was collected in the baseline year whereas sleepiness in the remote classes was collected during the pandemic year; ***Total score of PSQI (Pittsburgh sleep quality index) and their six components validated by Passos et al. (2017)^35^: subjective sleep quality (C1); sleep latency (C2); sleep duration (C3); habitual sleep efficiency (C4); sleep disturbances (C5); daytime dysfunction (C7); LMM = Linear mixed model; SD = Standard deviation; d = Cohen’s *d* effect size.

**Figure 1.**
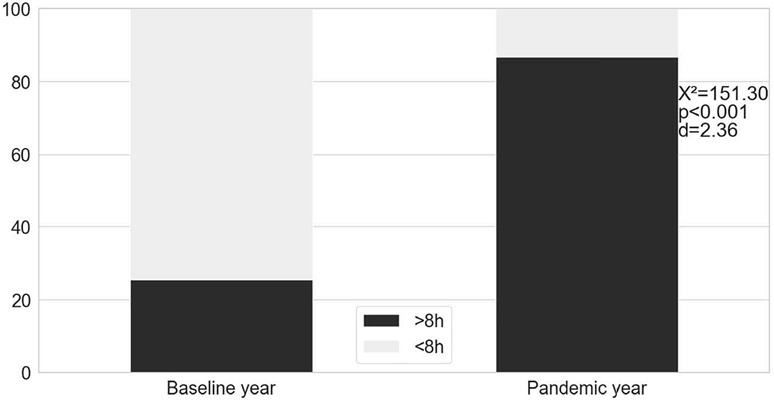
Comparison of the recommended time in bed between baseline and pandemic year. The bars indicate the percentage of the sample (n=259).

The sleep variables extracted from the PSQI are provided in Table 2. A bedtime delay of 01:16 hours (*p*<0.001, d=0.99) and later wake up times (*p*<0.001, d=3.37) were identifed during the pandemic year. During the pandemic, sleep latency was higher (*p*=0.03, d=0.19) and TIB was more than 2 hours longer (*p*<0.001, d=2.11) when compared to the baseline year. More than 80% of the students achieved the recommended TIB to adolescents (8-10 hours per night) during social isolation (*p*<0.001, d=2.36) considering that more than two-thirds of the sample slept less than the recommended in the prior year (Figure 1).

**Table 2.** Sleep variables extracted from the PSQI.

PSQI sleep variables	Baseline year	Pandemic year			LMM Fixed effects			
	Mean ± SD	Mean ± SD	Estimate	SD	t	p	d	95% CI
Bedtime	23:00 ± 01:12	00:16 ± 02:06	-0.05	0.01	-11.32	<0.001	0.99	-0.06 to -0.0
S. latency(min)	28.36 ± 32.61	35.54 ± 50.60	-6.09	2.78	-2.18	0.03	0.19	-11.55 to -0.6
Wake time	6:04 ± 00:32	9:52 ± 02:00	-0.16	0.01	-38.26	<0.001	3.37	-0.17 to -0.1
Time in bed	07:04 ± 01:10	09:36 ± 01:55	-151.85	6.35	-23.92	<0.001	2.11	-164.30 to -139

PSQI: Pittsburgh Sleep Quality Index. S. latency: Sleep Latency. LMM: Linear Mixed Model. SD: standard deviation. d: Cohen’s d effect size.

**Figure 2.**
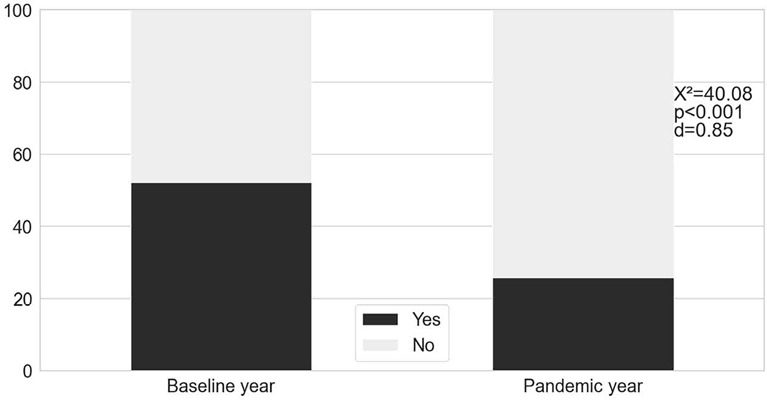
The percentage of adolescents that reported naps during weekdays (n=259).

The napping behavior changed during pandemic year: the frequency of nappers decreased during pandemic compared to the baseline year (*p*<0.001, d=0.85; Figure 2).

## DISCUSSION

This work contributed to understanding the magnitude of changes during the COVID-19 outbreak in sleep and napping habits among adolescents. The pandemic year played a key role in increasing TIB and the frequency of students reporting enough sleep duration. We found a reduction in both daytime sleepiness scored by PDSS and sleepiness in remote classes (compared to the sleepiness in the classroom before the pandemic year). A greater tendency toward eveningness together with reduction in the frequency of naps was found during the COVID-19 outbreak.

Recently, Liu et al. (2019)^37^ found benefts from the pandemic period to sleep health in preschoolers. Data collected during the COVID-19 lockdown in China (February 2020) showed later bedtimes and wake times, longer sleep duration, and shorter duration of naps, compared to the sample screened in 2018^37^. The sample was composed of children aged 4-6 years demonstrating that changes during the outbreak perceived in our adolescent sample could be extended to children’s sample. Furthermore, the sleep extension perceived in children and adolescents during lockdown is in line with previous studies that have found longer sleep duration during non-school days (e.g., holidays and vacation time)^37,38,39,40,41,42^.

Changes in sleep and napping among adolescents have been found following five subsequent weeks during lockdown. Later sleep onset times associated to an absence of differences in bedtimes and wake up times between weekdays and weekends were observed after the beginning of COVID-19 outbreak. The same study reported a substantial decrease in nap reports just after one week of the stay-at-home condition^8^. Similarly, Wright et al. (2020)^43^ found sleep health improvements in university students (mean age = 22.2 years) evaluated both in January and February, 2020 (before lockdown) and in April, 2020 (during lockdown). The difference in sleep duration between weekdays and weekends decreased during lockdown that may suggest a social jet lag reduction^43^. Additionally, sleep onset time became later as well as TIB and frequency of subjects that reported recommended sleep duration increased during COVID-19 outbreak. Gruber et al. (2020)^9^ found a similar scenario assessing sleep patterns in 45 adolescents (mean age 13.5 years) from Canada. Most of the adolescents reported sleep improvements during the pandemic. Similar to our findings, bedtimes and wake times were delayed for two hours. The students reported that they did not need to wake up earlier as one of the main reasons for this change^9^. Furthermore, 55% of adolescents attributed daytime sleepiness before the lockdown to the necessity to wake up early to get to school. Conversely, the low levels of daytime sleepiness experienced during the pandemic were associated to longer sleep times in 78% of the adolescents’ sampled^9^. These findings reinforce that early school starting times play an essential role to trigger chronic sleep restriction in adolescents.

The decrease of nap episodes has been recurrent in other recent investigations. During the pandemic, only 27.5% of Chinese preschoolers took naps during the week, compared to 79.8% that napped before this period^37^. The authors suggested that the absence of naps could be related to the lack of scheduled napping times commonly adopted by the Chinese education system for preschoolers^37^. Moreover, the follow-up study conducted by Roitblat et al. (2020)^8^ demonstrated a significant reduction in regular naps: from 421 adolescents reporting regular naps before lockdown, only 28 related napping behaviour two months after the beginning of lockdown.

The tendency toward eveningness could be explained by the greater sleep time autonomy during pandemic year. During lockdown, there was a change from an inappropriate early school start time during the baseline year to a flexible class time provided during lockdown (students could take asynchronous or recorded classes at any time). An opportunity has emerged: students could align their sleep patterns with their endogenous circadian time^44^. Furthermore, several studies have described an increasing eveningness preference from early to late adolescence^45,46,47,48^, which suggests an effect of additional age (baseline to pandemic year) to the tendency of eveningness in our sample.

This study has some limitations: the lack of an objective measurement of sleep (e.g., actigraphy) and also the assessment of mental health and daytime functioning during baseline and pandemic years. On the other hand, the major strength of this study is the longitudinal data collection that allowed us to identify critical changes in sleep and napping habits during COVID-19 outbreak.

In summary, our results help understand how much sleep and napping habits have changed during this atypical stay-at-home period. The sleep improvements observed during the COVID-19 outbreak reveal the inadequacy of early morning school start times. Adequate sleep duration is essential to mental performance and learning during this critical phase of life. A discussion about school time reschedule after COVID-19 outbreak is one of the key points of a movement toward a more inclusive educational system.

## References

[1] Aquino EML, Silveira IH, Pescarini JM, Aquino R, Souza-Filho JA, Rocha AS (2020). Social distancing measures to control the COVID-19 pandemic: potential impacts and challenges in Brazil. Ciênc Saúde Coletiva.

[2] Gualano MR, Lo Moro G, Voglino G, Bert F, Siliquini R (2020). Effects of COVID-19 lockdown on mental health and sleep disturbances in Italy. Int J Environ Res Public Health.

[3] Mônico-Neto M, Santos RVT, Antunes HKM (2020). The world war against the COVID-19 outbreak: don’t forget to sleep!. J Clin Sleep Med.

[4] Cellini N, Canale N, Mioni G, Costa S (2020). Changes in sleep pattern, sense of time and digital media use during COVID-19 lockdown in Italy. J Sleep Res.

[5] Figueiredo CS, Sandre PC, Portugal LCL, Mázala-de-Oliveira T, Chagas SL, Raony Í (2021). COVID-19 pandemic impact on children and adolescents’ mental health: biological, environmental, and social factors. Prog Neuro- Psychopharmacol Biol Psychiatry [Internet].

[6] Imran N, Zeshan M, Pervaiz Z (2020). Mental health considerations for children and adolescents in COVID-19 pandemic. Pak J Med Sci.

[7] Stern M, Wagner MH, Thompson LA (2020). Current and COVID-19 challenges with childhood and adolescent sleep. JAMA Pediatr.

[8] Roitblat Y, Burger J, Vaiman M, Nehuliaieva L, Buchris N, Shterenshis M (2021). Owls and larks do not exist: COVID-19 quarantine sleep habits. Sleep Med.

[9] Gruber R, Saha S, Somerville G, Boursier J, Wise MS (2020). The impact of COVID-19 related school shutdown on sleep in adolescents: a natural experiment. Sleep Med.

[10] Paraskakis E, Ntouros T, Ntokos M, Siavana O, Bitsori M, Galanakis E (2008). Siesta and sleep patterns in a sample of adolescents in Greece. Pediatr Int.

[11] Carskadon MA (2011). Sleep in adolescents: the perfect storm. Pediatr Clin North Am.

[12] Crowley SJ, Wolfson AR, Tarokh L, Carskadon MA (2018). An update on adolescent sleep: new evidence informing the perfect storm model. J Adolesc.

[13] Carvalho-Mendes RP, Dunster GP, Iglesia HO, Menna-Barreto L (2020). Afternoon school start times are associated with a lack of both social jetlag and sleep deprivation in adolescents. J Biol Rhythms.

[14] Singh R, Suri JC, Sharma R, Suri T, Adhikari T (2018). Sleep pattern of adolescents in a school in Delhi, India: impact on their mood and academic performance. Indian J Pediatr.

[15] Wahlstrom KL, Owens JA (2017). School start time effects on adolescent learning and academic performance, emotional health and behaviour. Curr Opin Psychiatry.

[16] Carskadon MA, Tarokh L (2014). Developmental changes in sleep biology and potential effects on adolescent behavior and caffeine use. Nutr Rev.

[17] Jakubowski K P, Hall MH, Lee L, Matthews KA (2016). Temporal relationships between napping and nocturnal sleep in healthy adolescents. Behav Sleep Med [Internet].

[18] McDevitt EA, Alaynick WA, Mednick SC (2012). The effect of nap frequency on daytime sleep architecture. Physiol Behav.

[19] Lo JC, Lee SM, Teo LM, Lim J, Gooley JJ, Chee MWL (2017). Neurobehavioral Impact of successive cycles of sleep restriction with and without naps in adolescents. Sleep.

[20] Ji X, Li J, Liu J (2019). The relationship between midday napping and neurocognitive function in early adolescents. Behav Sleep Med [Internet].

[21] Liu J, Feng R, Ji X, Cui N, Raine A, Mednick SC (2019). Midday napping in children: associations between nap frequency and duration across cognitive, positive psychological well-being, behavioral, and metabolic health outcomes. Sleep.

[22] Faraut B, Andrillon T, Vecchierini MF, Leger D (2017). Napping: a public health issue. From epidemiological to laboratory studies. Sleep Med Rev [Internet].

[23] Van Schalkwijk FJ, Sauter C, Hoedlmoser K, Heib DPJ, Klösch G, Moser D (2017). The effect of daytime napping and full-night sleep on the consolidation of declarative and procedural information. J Sleep Res.

[24] Nishida M, Walker M P (2007). Daytime naps, motor memory consolidation and regionally specific sleep spindles. PLoS One [Internet].

[25] Lau EYY, McAteer S, Leung CNW, Tucker MA, Li C (2018). Beneficial effects of a daytime nap on verbal memory in adolescents. J Adolesc.

[26] Slama H, Deliens G, Schmitz R, Peigneux P, Leproult R (2015). Afternoon nap and bright light exposure improve cognitive flexibility post lunch. PLoS One.

[27] Lo JC, Lee SM, Teo LM, Lim J, Gooley JJ, Chee MWL (2016). Neurobehavioral impact of successive cycles of sleep restriction with and without naps in adolescents. Sleep [Internet].

[28] MacDonald KJ, Lockhart HA, Storace AC, Emrich SM, Cote KA (2018). A daytime nap enhances visual working memory performance and alters event-related delay activity. Cogn Affect Behav Neurosci.

[29] Dhand R, Sohal H (2006). Good sleep, bad sleep! The role of daytime naps in healthy adults. Curr Opin Pulm Med [Internet].

[30] Suppiah HT, Low CY, Choong G, Chia M (2018). Effects of a short daytime nap on shooting and sprint performance in high-level adolescent athletes. Int J Sports Physiol Perform.

[31] Häusler N, Marques-Vidal P, Haba-Rubio J, Heinzer R (2019). Does sleep predict next-day napping or does napping infuence same-day nocturnal sleep? Results of a population-based ecological momentary assessment study. Sleep Med.

[32] Pereira ÉF, Carniel JD, Andrade RD, Pelegrini A, Anacleto TS, Louzada FM (2016). Translation and validation of the Pediatric Daytime Sleepiness Scale (PDSS) into Brazilian. J Pediatr (Rio J) [Internet].

[33] Carskadon MA, Vieira C, Acebo C (1993). Association between puberty and delayed phase preference. Sleep.

[34] Finimundi M, Barin I, Bandeira D, Souza DO (2012). Validação da escala de ritmo circadiano - ciclo vigília/sono para adolescentes. Rev Paul Pediatr.

[35] Passos MHP, Silva HA, Pitangui ACR, Oliveira VMA, Lima AS, Araújo RC (2017). Confabilidade e validade da versão brasileira do índice de qualidade do sono de Pittsburgh em adolescentes. J Pediatr (Rio J).

[36] Bertolazi AN, Fagondes SC, Hoff LS, Dartora EG, Miozzo ICS, Barba MEF (2011). Validation of the Brazilian Portuguese version of the pittsburgh sleep quality index. Sleep Med.

[37] Liu Z, Tang H, Jin Q, Wang G, Yang Z, Chen H (2020). Sleep of preschoolers during the coronavirus disease 2019 (COVID-19) outbreak. J Sleep Res.

[38] Warner S, Murray G, Meyer D (2008). Holiday and school-term sleep patterns of Australian adolescents. J Adolesc.

[39] Mak KK, Lee SL, Ho SY, Lo WS, Lam TH (2012). Sleep and academic performance in Hong Kong adolescents. J Sch Health.

[40] Blunden S, Magee C, Clarkson L, Searle A, Banks S, Olds T (2019). Interindividual and intraindividual variability in adolescent sleep patterns across an entire school term: a pilot study. Sleep Health.

[41] Harbard E, Allen NB, Trinder J, Bei B (2016). What’s keeping teenagers up? Prebedtime behaviors and actigraphy-assessed sleep over school and vacation. J Adolesc Health.

[42] Sousa IC, Louzada FM, Azevedo CVM (2009). Sleep-wake cycle irregularity and daytime sleepiness in adolescents on schooldays and on vacation days. Sleep Sci.

[43] Wright KP, Linton SK, Withrow D, Casiraghi L, Lanza SM, Iglesia H (2020). Sleep in university students prior to and during COVID-19 stay-at-home orders. Curr Biol.

[44] Marelli S, Castelnuovo A, Somma A, Castronovo V, Mombelli S, Bottoni D (2021). Impact of COVID-19 lockdown on sleep quality in university students and administration staff. J Neurol.

[45] Randler C (2011). Age and gender differences in morningness-eveningness during adolescence. J Genet Psychol.

[46] Randler C, Faßl C, Kalb N (2017). From lark to owl: developmental changes in morningness-eveningness from new-borns to early adulthood. Sci Rep [Internet].

[47] Kuula L, Pesonen AK, Merikanto I, Gradisar M, Lahti J, Heinonen K (2018). Development of late circadian preference: sleep timing from childhood to late adolescence. J Pediatr.

[48] Lehto JE, Aho O, Eklund M, Heinaro M, Kettunen S, Peltomäki A (2016). Circadian preferences and sleep in 15- to 20-year old Finnish students. Sleep Sci.

